# Orphan Nuclear Receptors TR2 and TR4 in Erythropoiesis: From Mechanisms to Therapies

**DOI:** 10.3390/biom15060798

**Published:** 2025-05-31

**Authors:** Yunlong Liu, Helian Yang, Mengtian Ren, Qing Yu, Qingyang Xu, Xiuping Fu

**Affiliations:** School of Life Sciences and School of Chemistry, Tiangong University, Tianjin 300387, China; 2431131415@tiangong.edu.cn (H.Y.); mengtianren@tiangong.edu.cn (M.R.); 2313920214@tiangong.edu.cn (Q.Y.); 2313920225@tiangong.edu.cn (Q.X.)

**Keywords:** orphan nuclear receptors TR2/TR4, erythropoiesis, DRED complex, β-thalassemia, sickle cell disease, hematological disorder therapy

## Abstract

Testicular orphan receptors TR2 and TR4 serve as central regulators of erythropoiesis, orchestrating the entire continuum of erythroid progenitor cell proliferation, differentiation, and maturation. As core components of the direct repeat erythroid determinant (DRED) complex, they activate erythroid-specific transcriptional programs to dynamically control the spatiotemporal expression of globin genes. These nuclear receptors not only engage in functional interactions with key erythroid transcription factors GATA1 and KLF1 to coregulate erythroid differentiation and maturation but also recruit epigenetic modifier complexes such as DNMT1 and LSD1 to modulate chromatin states dynamically. Research has established that dysfunctions in TR2/TR4 are implicated in β-thalassemia and sickle cell disease (SCD): β-thalassemia is associated with the defective silencing of γ-globin genes, while in SCD, TR2/TR4 antagonizes BCL11A to reactivate fetal hemoglobin (HbF) expression. This review systematically dissects the molecular regulatory networks of TR2/TR4 in erythroid cells, interprets their dual regulatory properties across different stages of erythroid differentiation, and explores the therapeutic potential of targeting TR2/TR4 for treating erythroid-related disorders such as β-thalassemia and SCD, thereby providing novel directions for hematological disorder therapy.

## 1. Introduction

Erythrocytes, as critical functional units of the human circulatory system, play a central role in gas transport (oxygen O_2_/carbon dioxide CO_2_) and nutrient distribution. These highly specialized anucleate cells constitute the predominant cellular component in blood, accounting for approximately 99.9% of total blood cells [1,2,3]. Their physiological function relies on the molecular mechanism of hemoglobin for oxygen-specific binding and release [4]. Globins, the core functional subunits of hemoglobin, undergo strict developmental stage-specific isoform switching: a sequential expression pattern spanning embryonic (ζ/ε chains), fetal (α/γ chains), and adult (α/β chains) stages, generating functionally distinct hemoglobin variants adapted to varying oxygen environments [5]. This process is orchestrated through coordinated regulation by transcription factors (e.g., GATA1 and BCL11A), epigenetic regulatory complexes (e.g., DRED complex), and three-dimensional chromatin architecture [6,7,8]. Dysregulation of this switching process underlies the pathogenesis of hemoglobinopathies such as β-thalassemia and SCD [9].

Testicular orphan receptors TR2 (NR2C1) and TR4 (NR2C2), members of the NR2C subfamily of nuclear receptors, function as the DNA-binding core of the DRED complex and have emerged as pivotal regulators in erythroid biology [10,11]. The investigation of their roles originated in the late 20th century during comprehensive explorations of nuclear receptor family functionalities. Nuclear receptors are recognized as master regulators of gene expression, playing essential roles in cellular differentiation, development, and metabolism [12]. Studies demonstrate that TR2/TR4 govern the entirety of erythropoiesis through multi-tiered regulatory networks, encompassing critical processes such as progenitor cell proliferation, terminal differentiation, and hemoglobin synthesis [13].

Evolutionarily, TR2 and TR4 constitute a unique dimeric subgroup within the nuclear receptor superfamily. Sequence alignment reveals 82% homology in their DNA-binding domains (DBDs) and 65% homology in ligand-binding domains (LBDs), significantly exceeding their evolutionary divergence from other nuclear receptors (e.g., AR and ER; DBD < 50% and LBD < 10%) [14]. This structural distinctiveness facilitates preferential heterodimerization between TR2 and TR4, enabling their incorporation into the 540 kDa DRED complex. As integral components of DRED [11], they mediate precise regulation of erythroid development and maturation by recognizing direct repeat (DR) AGGTCA sequences with variable nucleotide spacers, thereby orchestrating spatiotemporal control of target gene expression [15].

During erythropoiesis, TR2/TR4 exhibit dual regulatory properties: they mediate gene silencing through the recruitment of epigenetic modifier complexes (e.g., HDACs/DNMTs) while simultaneously activating differentiation-associated gene expression via chromatin remodeling [16]. This dynamic equilibrium is critical for hemoglobin switching (e.g., γ-to β-globin transition) and erythrocyte membrane skeleton assembly. Notably, the TR2/TR4-mediated regulatory mechanism of HbF expression has unveiled novel therapeutic avenues for SCD and β-thalassemia [17]. In SCD models, TR4 antagonizes BCL11A-driven transcriptional repression, elevating HbF levels by over 40% and significantly ameliorating the sickling phenotype [18]. Breakthroughs in drug development targeting this pathway are underway, with multiple TR2/TR4-specific small-molecule modulators currently in preclinical evaluation [19,20]. Furthermore, recent identification of TR2/TR4-selective transcriptional coregulators has expanded opportunities for developing inhibitors to treat erythroid-related disorders linked to these nuclear receptors [21,22].

In this review, we comprehensively synthesize recent advances in the study of nuclear receptors TR2/TR4 within erythroid biology, summarizing current structural foundations underlying their functional mechanisms. We systematically dissect the intricate regulatory networks orchestrated by TR2/TR4 during erythroid development, maturation, and hemoglobin expression, while critically evaluating their pathophysiological roles in erythrocyte disorders such as SCD and β-thalassemia. Additionally, we explore emerging therapeutic strategies targeting TR2/TR4, including the development of receptor-specific agonists and inhibitors. These findings are anticipated to deepen the mechanistic understanding of TR2/TR4 in erythropoiesis, erythrocyte maturation, and disease pathogenesis, thereby establishing a scientific rationale for precision therapeutics in hemoglobinopathies.

## 2. Structures of TR2/TR4

**Figure 1:**
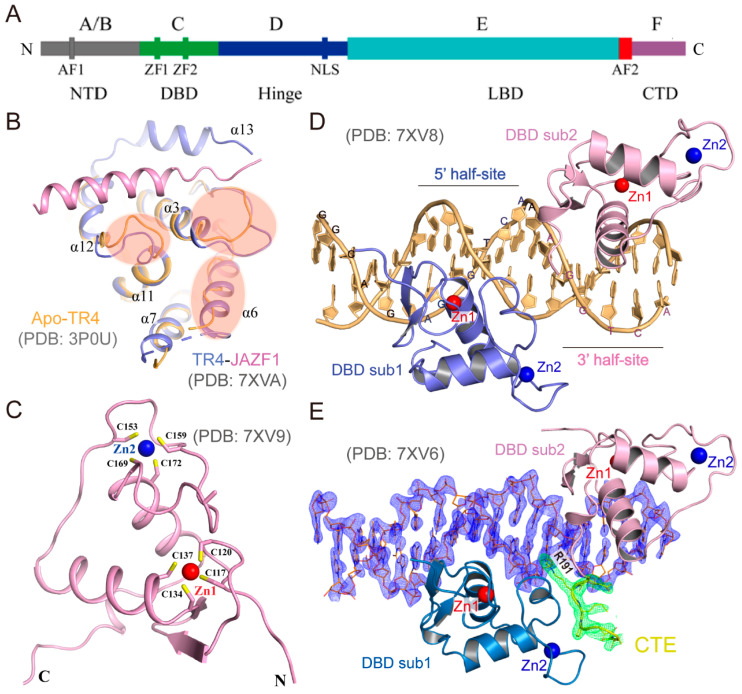
Overall structures of the TR4 [29,30]. (**A**) The schematic diagram of the multi-domain structure of TR2/TR4. (**B**) Structural overlay of apo-TR4 LBD (PDB 3P0U, orange) and the TR4_LBD_–JAZF1 complex (PDB 7XVA, purple and pink) shows the conformational changes in the TR4 LBD caused by JAZF1 binding. (**C**) Overall structure of TR4 DBD (PDB 7XV9). (**D**) Structure of the TR4_DBD_–dsDNA complex (PDB 7XV8). (**E**) Structure of the TR4_DBD-CTE_–dsDNA complex (PDB 7XV6). The CTE of TR4 DBD is colored yellow. The composite simulated-annealing *Fo–Fc* ‘omit’ electron density maps of CTE and dsDNA are shown at 2.5 σ.

TR2 and TR4, critical members of the orphan nuclear receptor superfamily, were first cloned in 1989 and 1994, respectively [14,23,24]. Like most nuclear receptors, TR2/TR4 exhibit the canonical modular architecture of the nuclear receptor family, comprising a DBD, an LBD, and transcriptional activation function (AF) domains [25,26,27]. As depicted in Figure 1A, their modular structure includes the following:

A/B region: A less conserved region harboring an N-terminal AF-1 domain, which interacts with promoter-specific coregulators to activate transcription.

C region (DBD): A conserved central domain containing two zinc finger motifs that direct the nuclear receptors to highly specific DNA sequences containing cognate response elements.

Hinge region: A flexible and least conserved segment connecting the DBD and LBD, ensuring receptor flexibility and encoding nuclear localization signals (NLS).

D region (LBD): A highly conserved domain adopting the classical α-helical sandwich structure. Variations in the hydrophobic ligand-binding pocket enable nuclear receptors to discriminate between diverse ligands. Ligand binding induces conformational changes in the AF-2 helix, facilitating interactions with coregulators. Notably, despite lacking identified endogenous ligands, orphan nuclear receptors such as TR2/TR4 retain these domains, suggesting latent functional diversity and potential ligand-binding capabilities [28].

F region: Beyond its previously identified role in mediating JAZF1 binding to suppress coactivator recruitment [29], this hypervariable C-terminal domain retains predominantly uncharacterized functions and is notably absent in select nuclear receptor family members.

The structural architecture of TR4’s key functional domains has been elucidated. Zhou et al. determined the LBD structure of TR4 (Figure 1B, orange, PDB 3P0U) using X-ray crystallography at a resolution of 3.00 Å, revealing its auto-inhibited conformation in the ligand-free state [30]. In this conformation, the ligand-binding pocket is occluded by the C-terminal α10 helix, while the coactivator-binding site is sterically occupied by the AF-2 helix, thereby preventing ligand-independent activation of the receptor. In 2023, we further resolved a high-resolution (1.86 Å) crystal structure of the TR4 LBD (Figure 1B, purple, PDB 7XVA) in complex with the JAZF1 [29] (Figure 1B, pink). This breakthrough unveiled electron density for a previously unobserved α6 helix, a unique α13 helix, and residues 446–454 located between the α5 and α7 helices, which were absent in the apo-TR4 LBD structure. Structural analyses demonstrated that JAZF1 suppresses TR4 transcriptional activity via a novel inhibitory mechanism: it binds to a previously unidentified surface pocket on TR4 and stabilizes the α13 helix, a structural motif unique to TR2/TR4 within the nuclear receptor superfamily. This discovery provides critical structural insights into TR4’s roles in diverse physiological and pathological processes and informs rational drug design targeting TR4-specific interactions.

To further elucidate the molecular mechanisms underlying TR4-mediated target gene recognition, we employed X-ray crystallography to resolve the Holo-TR4 DBD structure (Figure 1C, PDB 7XV9), as well as the TR4_DBD_-DNA (Figure 1D, PDB 7XV8) and TR4_DBD-CTE_-DNA (Figure 1E, PDB 7XV6) complex structures [29]. In the resolved structures, the TR4 DBD adopts two C4-type zinc finger motifs, with DNA recognition primarily mediated by the α-helix of the first zinc finger. Structural analyses revealed that TR4 engages target DNA through multiple mechanisms, including direct or water-mediated hydrogen bonding, DNA shape readout, and cooperative DBD dimerization. Furthermore, DNA mutant binding assays identified the TR4 target sequence as a double-stranded DNA (dsDNA) motif containing two direct repeat PuGGTCA hexanucleotide half-sites (Pu: purine), with the 5′ half-site playing a dominant role in TR4 binding. TR4 exhibits higher binding affinity for DR5 sequences and forms stable heterotrimeric complexes with DR1 sequences. Microscale Thermophoresis (MST) binding assays further demonstrated that the TR4 DBD and hinge domain are involved in DNA interaction, whereas the NTD and LBD do not directly participate in DNA binding [29].

Despite the absence of structural data for TR2, key residues critical for DNA recognition and coregulator binding are conserved between TR2 and TR4 [29], suggesting that they share analogous structural frameworks for target gene recognition and transcriptional coregulator recruitment. These structural insights provide mechanistic validation for TR2/TR4-mediated DNA recognition and coregulator recruitment during transcriptional regulation of critical genes in erythropoiesis.

## 3. TR2/TR4 in Erythropoiesis

### 3.1. Erythrocyte Differentiation and Globin Switching

**Figure 2:**
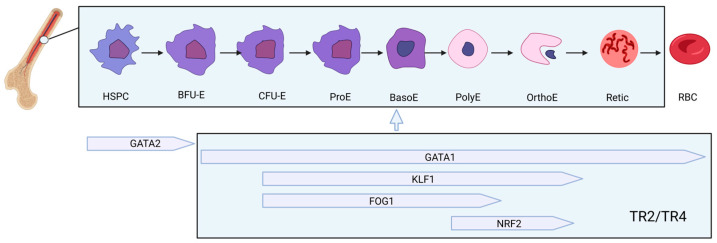
Overview of developmental stages and transcriptional regulation of erythropoiesis. Created by BioGDP.com [33].

Human erythropoiesis is a tightly regulated, multistage process that originates from hematopoietic stem and progenitor cells (HSPCs) in the bone marrow and culminates in the formation of mature red blood cells (RBCs) [31,32]. During this differentiation cascade, multipotent progenitors first give rise to megakaryocyte-erythroid progenitors (MEPs), which then commit to the erythroid lineage through the sequential emergence of burst-forming unit-erythroid (BFU-E) and colony-forming unit-erythroid (CFU-E) cells. These early progenitors, collectively referred to as erythroid progenitors (EPs), transition into morphologically identifiable precursors beginning with proerythroblasts (ProE), followed by basophilic (BasoE), polychromatic (PolyE), and orthochromatic erythroblasts (OrthoE). This maturation phase is marked by progressive nuclear condensation and culminates in enucleation, producing reticulocytes (Retic), which are released into circulation and mature into fully functional RBCs (Figure 2).

The differentiation trajectory is orchestrated by a dynamic network of transcription factors (TFs) that exhibit stage-specific expression and regulatory functions (Figure 2). Currently, there is no definitive evidence demonstrating that TR2/TR4 are involved in the regulation of the early stage in MEPs. However, substantial evidence indicates that TR2/TR4 play a critical role during the intermediate to late stages of erythropoiesis (BFU-E→RBC) by repressing the expression of GATA1, a key regulator of erythroid development, and by modulating or recruiting multiple erythropoiesis-related transcriptional regulators such as KLF1, FOG1, and NRF2 [10,11,13,14,16]. Notably, although partial overlap in their activities is also observed, these TR2/TR4-coordinated regulatory factors function at distinct stages of erythroid development (Figure 2). Specifically, as differentiation proceeds, GATA1 expression rises and actively represses GATA2, establishing a transcriptional switch critical for erythroid commitment. GATA1 [34,35,36], in concert with cofactors such as FOG1 and the NuRD complex, orchestrates the activation of erythroid-specific genes while repressing alternate lineage programs. KLF1 (EKLF) emerges as a key regulator from the ProE stage onward, driving terminal differentiation by promoting hemoglobin synthesis, chromatin condensation, and cell cycle exit. FOG1, a GATA1 cofactor, plays essential roles throughout erythroid development, modulating chromatin architecture and transcriptional output [37]. In the later stages, NRF2 becomes increasingly important, regulating antioxidant responses and contributing to cellular resilience during enucleation and reticulocyte maturation [38]. Together, these transcription factors function in a coordinated, temporally controlled manner to ensure proper erythroid lineage progression, gene expression fidelity, and red cell functionality.

**Figure 3:**
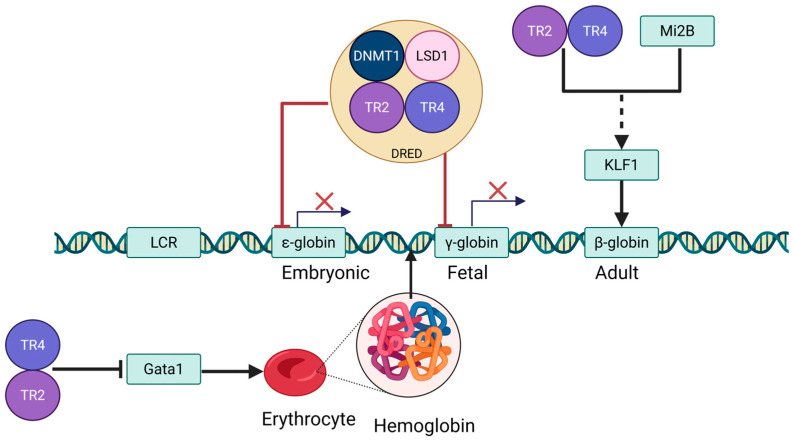
TR2/TR4-mediated spatiotemporal regulation of globin gene expression. Symbol ×: the transcription inhibition of globin protein. Created by BioGDP.com [33].

Erythropoiesis involves two developmental switches in globin gene transcription [39] (Figure 3). The first switch silences embryonic ε-globin (HBE1) and activates fetal γ-globin (HBG1/2), a process initiated in primitive erythroid progenitors from the yolk sac. This switch marks the shift in erythropoiesis from the yolk sac to the fetal liver, where fetal hemoglobin (HbF) takes over to meet the oxygen demands of the developing fetus [40]. Around birth, a second switch occurs, transitioning from γ-globin to adult β-globin (HBB) as hematopoiesis shifts to the bone marrow, and adult hemoglobin becomes the predominant form [41]. The molecular events regulating these switches share common epigenetic mechanisms, such as histone modifications and DNA methylation, as well as interactions with the Locus Control Region (LCR) [42,43]. However, the transcription factors involved are distinct: the embryonic-to-fetal switch is driven by GATA1/2, FOG-1, and NF-E2, while the fetal-to-adult switch is primarily regulated by BCL11A, SOX6, and KLF1 [44]. These differences reflect the specific roles each switch plays during development, with the embryonic-to-fetal switch occurring early on and the fetal-to-adult switch postnatally, ensuring precise regulation of hemoglobin synthesis at different stages of life (Figure 3).

### 3.2. Role of TR2/TR4 in Erythropoiesis

TR2 and TR4 are structurally related orphan nuclear receptors with overlapping but distinct roles in erythropoiesis. Both bind DR1 DNA elements in a ligand-independent manner and recruit co-regulators (e.g., SRC/p160 family) to modulate erythroid gene expression [45]. However, their activation pathways and functional roles differ significantly. TR4 is activated by EPO/STAT5 signaling and endogenous prostaglandins, playing a key role in early erythropoiesis (MEP→CFU-E transition) [46]. It recruits NCoA1/SRC1 to enhance erythroid gene expression (e.g., β-globin) and activates GATA1 [10,45]. In contrast to TR4, which plays a dominant role in erythroid differentiation, TR2 generally acts as a co-regulator that enhances TR4 function during erythropoiesis. Although TR2 alone exerts limited effects and its deletion produces only mild phenotypes [10], it may play a more distinct role during late-stage maturation (OrthoE→RBC), potentially through interaction with DAX1 to modulate oxidative stress responses. In this context, TR2 has been implicated in activating Nrf2-dependent antioxidant pathways, which help protect erythrocytes from oxidative damage [47,48]. Additionally, in certain cancer-related pathways, TR2 responds to Wnt/β-catenin signaling and synthetic agonists [49,50], suggesting a broader regulatory capacity that extends beyond its traditional role in erythropoiesis.

As core components of the DRED complex [10,11], TR2/TR4 orchestrate the spatiotemporal-specific expression of globin genes by binding DR1 elements as heterodimers [16,51,52,53,54] (Figure 3). These receptors dynamically regulate chromatin states through recruitment of epigenetic corepressor complexes, including DNA methyltransferase 1 (DNMT1), nucleosome remodeling and deacetylase (NuRD), lysine-specific histone demethylase 1 (LSD1)/corepressor of REST (CoREST), and HDAC3, which mediate nucleosome remodeling, histone deacetylation, and H3K4 demethylation [55]. Concurrently, TR2/TR4 functionally interact with erythroid master transcription factors GATA1, GATA2, and KLF1 to cooperatively regulate erythroid differentiation and maturation [42]. For instance, during embryogenesis, TR2-TR4 heterodimers bind promoters of ε-globin and γ-globin genes to enforce transcriptional silencing [13], whereas in adult erythrocytes, they selectively target embryonic β-type globin (HBBP1) promoters to maintain repression via epigenetic modifications [56]. Notably, TR4 exhibits dual regulatory roles in erythroid maturation: Its restricted expression downregulates heme biosynthesis genes (e.g., ALAS2) while activating the proliferation suppressor *CDKN1C*, highlighting its precise control over the balance between erythroblast proliferation and terminal differentiation [52].

The regulatory network of TR2/TR4 spans the entire erythroid developmental continuum. During the embryonic stem cell stage, these receptors govern pluripotency gene expression by binding DR1 elements within the Oct3/4 promoter region and interact with the retinoic acid signaling pathway to modulate stem cell fate determination [57,58]. In the terminal differentiation phase, the DRED complex suppresses ε- and γ-globin transcription via high-affinity binding to DR1 sequences, with its dysfunction directly linked to aberrant γ-globin reactivation in hereditary persistence of fetal hemoglobin (HPFH) syndrome [51]. Point mutations on DR1 sequences impair TR2/TR4 binding capacity, identifying these loci as potential therapeutic targets for SCD.

Both gain- and loss-of-function studies consistently demonstrate that TR2 and TR4 are essential regulators of erythropoiesis, rather than peripheral modulators of globin gene switching [10,11,13,51,52]. Forced erythroid-specific overexpression of TR2 and/or TR4 in transgenic mice resulted in transient embryonic anemia (10.5–13.5 dpc), marked by pale fetal livers, reduced numbers of primitive erythroid progenitors, and impaired hemoglobinization—particularly pronounced in TgTR2/TR4 embryos [10]. Although this early defect was resolved by 15.5 dpc and adult hematological parameters were normalized, it underscores the sensitivity of primitive erythropoiesis to TR2/TR4 dosage. Conversely, conditional deletion of both TR2 and TR4 in adult bone marrow progenitors led to severely impaired erythroid differentiation, downregulation of KLF1, upregulation of GATA1, and reactivation of embryonic and fetal globin genes alongside suppression of adult β-globin expression [13,51,52]. These alterations were absent in single knockouts, emphasizing the redundant and compensatory functions of TR2 and TR4. Collectively, these findings establish TR2/TR4 as central components of the erythroid transcriptional network, required for both early lineage commitment and developmental regulation of globin gene expression.

In addition to TR2 and TR4, DNMT1 and LSD1 function as cofactors to assemble the tetrameric core DRED complex [16] (Figure 3). DNMT1, previously characterized as a maintenance DNA methyltransferase, recognizes and methylates CpG dinucleotides positioned on the complementary strand of methylated CpG (MeCpG) sites, thereby preserving stable DNA methylation patterns in the genome following DNA replication [59]. LSD1 catalyzes the removal of methyl groups from mono- and dimethylated histone H3 lysine 4 (MeH3K4 and Me2H3K4, respectively), which are established epigenetic signatures marking transcriptionally active genes [60]. This core repressive tetramer (TR2-TR4-DNMT1-LSD1) further interacts with histone deacetylases 1/2 (HDAC1/2), NuRD, CoREST, TRIM28 (TIF1), and HDAC3 to form larger multifunctional complexes [16]. This multi-tiered epigenetic regulatory network plays a critical physiological role in erythroid development. Through dynamic recruitment of chromatin remodelers such as HDACs and NuRD, the DRED complex orchestrates precise spatiotemporal regulation of erythroid-specific genes, including the β-globin gene cluster, by modulating their expression patterns [61].

## 4. TR2/TR4 in Hemoglobin Expression

Hemoglobin (Hb) is a vital protein in erythrocytes, consisting of two α-subunits and two β-subunits that assemble into a tetrameric structure [62,63]. Its primary function is the transport of oxygen and carbon dioxide [64,65,66]. Each subunit contains a heme group, which is the critical moiety enabling oxygen binding [67,68,69]. TR2/TR4 regulate hemoglobin homeostasis through a dual mechanism: (1) physiological regulation via modulation of erythroid-specific transcription factors such as GATA1, thereby influencing erythroid differentiation [10,45,70], and (2) pathological intervention by suppressing γ-globin expression to mediate hemoglobin subtype switching [11,53,71,72,73] (Figure 3).

During embryonic and fetal development, hemoglobin is predominantly composed of two α-subunits and two γ-subunits, forming HbF [39]. Postnatally, this transitions to adult hemoglobin (HbA), characterized by β-subunits replacing γ-subunits. TR2/TR4 play a central role in this developmental switch, exerting both direct and indirect control over globin gene regulation. Evidence from overexpression [10,11] and knockout [13,42,51,52] studies indicates that TR2/TR4 directly repress embryonic and fetal globin genes (such as ey, ε, and γ) through recognition of DR elements within these loci, likely via the DRED complex. This is supported by increased primary transcript levels of ε- and γ-globins in TR2/TR4-deficient fetal liver, confirming transcriptional derepression. These findings highlight a direct role for TR2/TR4 in silencing non-adult globin genes to facilitate the transition toward definitive erythropoiesis.

In contrast, the reduction in adult β-globin expression observed in TR2/TR4 knockout models appears to be an indirect consequence of altered transcriptional networks. The β-globin locus lacks DR motifs, and no evidence supports direct TR2/TR4 binding. Instead, TR2/TR4 deletion disrupts expression of upstream regulators such as KLF1 [42,74] and GATA1 [10], which modulate BCL11A—a critical activator of adult β-globin and repressor of fetal globin genes. Thus, β-globin downregulation is likely mediated through a KLF1–BCL11A-dependent pathway [75]. Collectively, these data support a model in which TR2/TR4 act as developmental repressors of embryonic and fetal globin genes, while indirectly influencing adult globin expression via broader erythroid transcriptional regulation.

### 4.1. Coordinated Regulation of TR2/TR4 and GATA

GATA transcription factors (GATA1/2/3), members of the zinc finger protein family, are DNA-binding regulators that recognize the WGATAR motif to control erythroid-specific gene expression [76,77,78,79,80]. Among them, GATA1 and GATA2 function as master regulators at distinct stages of erythropoiesis, with GATA1 being indispensable for terminal erythroid differentiation. GATA1 not only activates erythropoietin (EPO) signaling to ensure erythroid precursor survival, but also orchestrates the expression of key erythroid genes, including β-globin [81,82,83]. However, GATA1 activity must be precisely modulated—both deficiency and overexpression can disrupt erythroid maturation and cause developmental arrest [10]. During the final stages of differentiation, GATA1 expression naturally declines, although the mechanism governing this repression was previously unclear.

Recent studies have elucidated that TR2 and TR4, orphan nuclear receptors of the nuclear receptor superfamily, maintain erythroid differentiation homeostasis in part by repressing Gata1 transcription [45,70]. These receptors, expressed in erythroid cells, form homo- or heterodimers that constitute the DNA-binding scaffold of the DRED complex. TR2/TR4 bind to evolutionarily conserved direct repeat (DR) elements within the GATA1 hematopoietic enhancer (G1HE), located approximately 3.7 kb upstream of the Gata1 1b exon [10]. This interaction has been confirmed both in vitro and in vivo, including via ChIP assays in human erythroblasts, and has functional consequences: forced expression of TR2/TR4 in transgenic mice leads to reduced GATA1 levels and transient embryonic anemia, while knockout or RNAi-mediated depletion of TR2/TR4 results in elevated GATA1 transcript levels [10]. These findings underscore that TR2/TR4 act as direct upstream repressors of GATA1, modulating its expression in a context- and stage-specific manner during terminal erythroid maturation (Figure 3).

### 4.2. Coordinated Regulation of TR2/TR4 and KLF1

Loss of TR2 and TR4 in bone marrow progenitors results in a marked downregulation of KLF1 [74], a pivotal erythroid transcription factor required for the activation of adult β-globin and the repression of fetal γ-globin expression [74,75]. KLF1 expression is transcriptionally regulated by GATA-1, positioning it downstream in a regulatory cascade essential for definitive erythropoiesis [74]. Functional studies have shown that KLF1 directly activates BCL11A, a major silencer of γ-globin [75], while also modulating the expression of KLF3 and KLF8—transcriptional repressors implicated in the silencing of embryonic globin genes. Mice deficient in both KLF3 and KLF8 exhibit derepression of embryonic globin, underscoring their collaborative role in globin gene silencing [42,84]. Notably, haploinsufficiency or loss-of-function mutations in KLF1, such as those identified in HPFH, lead to impaired BCL11A expression and elevated HbF levels in adults.

Importantly, TR2 and TR4—core DNA-binding components of the DRED repressor complex—may influence KLF1 both directly and indirectly. Emerging evidence indicates that these orphan nuclear receptors, in addition to repressing Gata1 during late erythroid differentiation [10], functionally associate with Mi2B (CHD4), a key component of the NuRD chromatin remodeling complex. Mi2B is known to positively regulate KLF1 and BCL11A expression and serves as a cofactor of TR2/TR4 within DRED. This suggests that TR2/TR4 may participate in the hierarchical regulation of KLF1 expression through their interaction with Mi2B, thereby influencing downstream β-type globin gene switching [42,85] (Figure 3). In this context, the DRED complex not only represses fetal and embryonic globin genes directly but may also facilitate chromatin remodeling events that reinforce the adult globin expression program through modulation of the KLF1–BCL11A axis.

### 4.3. TR2/TR4 in the Regulation of Heme Biosynthesis

**Figure 4:**
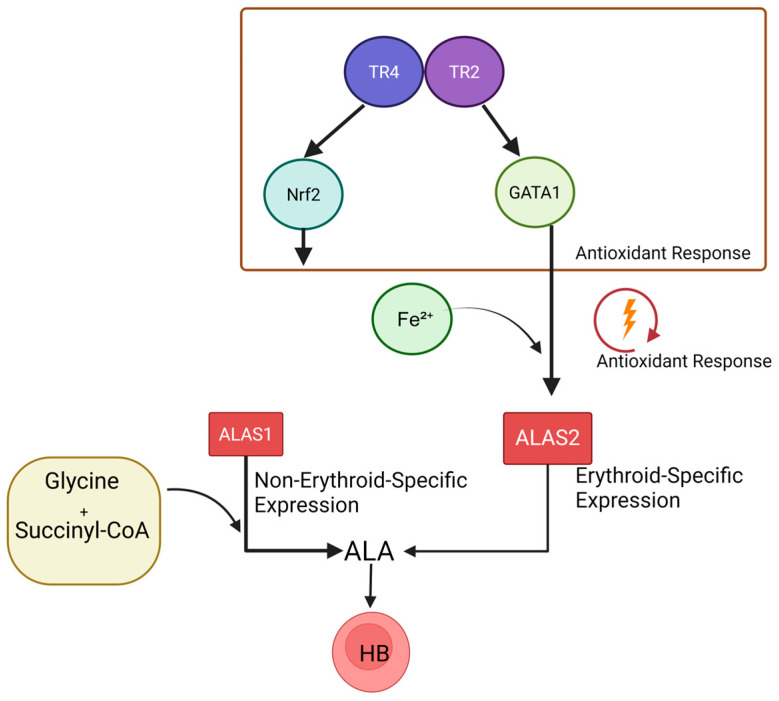
TR2/TR4-mediated regulatory pathway in heme biosynthesis. Created by BioGDP.com [33].

5-Aminolevulinic acid synthase (ALAS), a homodimeric pyridoxal 5′-phosphate (PLP)-dependent enzyme, is often termed the “gatekeeper” of heme biosynthesis [86,87]. In mammals, ALAS exists as two functionally distinct isoforms: ALAS1, a ubiquitously expressed constitutive isoform regulated by heme-mediated negative feedback via its mRNA 5′UTR [88], and ALAS2, an erythroid-specific isoform dedicated to hemoglobin synthesis. ALAS2 expression is modulated by erythroid differentiation-associated transcription factors (e.g., GATA1 [89,90,91], Nrf2 [92]) and reactive oxygen species (ROS) levels [93], with Fe^2+^ availability acting as a primary regulatory signal [94]. TR2/TR4 likely influence this pathway through two mechanisms: (1) coordinating with Nrf2 to regulate antioxidant response elements (AREs), maintaining redox balance in erythroid precursors, and (2) indirectly modulating ALAS2 transcription via the GATA1 axis to synchronize heme synthesis with hemoglobin assembly (Figure 4). Notably, dysregulation of the TR2/TR4 network may exacerbate pathological outcomes in congenital erythropoietic porphyria caused by ALAS2 deficiency [95,96]. This underscores the dual role of TR2/TR4 not only in physiological heme synthesis but also as potential therapeutic targets for heme metabolism disorders.

Recent studies have revealed a complex regulatory network through which TR2/TR4 modulate the expression of the *ALAS2* gene. When the abundance of TR4 is reduced alone, ALAS2 expression decreases significantly—by approximately 3.6-fold [13]. This reduction results from the loss of TR4’s direct transcriptional activation, as TR4 is known to bind the ALAS2 enhancer and recruit coactivators such as NCoA1/SRC1 [13]. In addition, TR4 deficiency leads to a downregulation of KLF1, a critical activator of ALAS2, further contributing to the suppression of ALAS2 expression [74]. Interestingly, when the abundance of TR2/TR4 is reduced, ALAS2 expression is paradoxically upregulated. This apparent contradiction can be explained by the role of the TR2/TR4 complex in repressing GATA1. Dual deletion removes this repression, increasing GATA1 expression [10]. GATA1, a master regulator of erythroid development, is capable of directly activating ALAS2 [89], thereby overriding the loss of TR4-mediated activation. This bidirectional regulatory mechanism highlights the intricate balance within the erythroid transcriptional network. TR4 maintains basal ALAS2 expression through both direct enhancer binding and indirect support via KLF1, while TR2, in cooperation with TR4, modulates ALAS2 indirectly by controlling GATA1 levels.

## 5. TR2/TR4 in Erythrocyte Disorders

The core pathogenesis of β-hemoglobinopathies (encompassing SCD and β-thalassemia) stems from β-globin chain dysfunction: β-thalassemia manifests through insufficient β-chain synthesis [97], whereas SCD arises from β-chain mutations (HbS) that induce erythrocyte sickling [98]. Potential therapeutic strategies for these disorders focus on reversing the dysregulated postnatal hemoglobin switching process—during normal development, the DRED complex mediates fetal-to-adult hemoglobin transition (from HbF [α2γ2] to HbA [α2β2]) through binding of nuclear receptor TR2/TR4 heterodimers to γ-globin (HBG) gene promoters, followed by recruitment of corepressors such as BCL11A to enforce HBG epigenetic silencing [99,100]. Elucidating this regulatory axis has identified critical therapeutic targets: Targeted disruption of the TR2/TR4-BCL11A regulatory complex can relieve HBG suppression, elevating HbF levels to 15–30% [101]. This compensates for β-chain deficiencies or inhibits HbS polymerization, thereby significantly improving erythrocyte functionality.

### 5.1. TR2/TR4 in SCD

SCD is caused by a mutation in the HBB gene where glutamic acid is substituted by valine at the sixth codon position [102], leading to the polymerization of abnormal hemoglobin HbS within erythrocytes [103]. This pathological aggregation distorts erythrocytes into a sickle-shaped morphology [104], triggering hallmark clinical features such as chronic hemolysis and vaso-occlusive complications [105]. Critical studies reveal that maintaining HbF levels above 8.6% in SCD patients effectively suppresses the elongation of sickle hemoglobin polymers and is associated with a significant reduction in mortality rates [106,107].

Despite the well-established role of TR2/TR4 heterodimers in repressing γ-globin expression under physiological conditions, their regulatory behavior in disease models exhibits unexpected complexity. Paradoxically, forced overexpression of TR2/TR4 in adult murine erythrocytes was observed to aberrantly reactivate fetal γ-globin genes [71]. In humanized SCD murine models, this intervention elevated the HbF/total hemoglobin ratio from 7.6% to 18.6%, concurrently improving hematocrit levels from 23% to 34% and reducing reticulocyte proportions from 61% to 18% [71], collectively indicating marked attenuation of hemolytic activity. Furthermore, TR2/TR4 overexpression alleviated pathological manifestations, including hepatosplenomegaly, hepatic parenchymal necrosis, and systemic inflammatory responses. These counterintuitive findings suggest that TR2/TR4 may indirectly modulate γ-globin expression through dynamic feedback mechanisms or context-dependent interactions with developmental regulators such as GATA1 [10,45,70].

Therapeutic strategies targeting HbF induction face limitations with hydroxyurea (HU), the only FDA-approved agent, which operates via ribonucleotide reductase inhibition to induce cell cycle arrest. However, its clinical efficacy is variable and often compromised by dose-dependent myelosuppression [108,109,110,111,112]. In contrast, interventions targeting the TR2/TR4-BCL11A regulatory axis offer distinct mechanistic advantages: Direct inhibition of TR2/TR4 disrupts γ-globin transcriptional silencing, while calibrated modulation of TR2/TR4 expression levels may induce sustained HbF elevation through indirect epigenetic reprogramming pathways [101]. This dual approach holds promise for developing curative therapies that circumvent the need for chronic drug administration, potentially revolutionizing the management of β-hemoglobinopathies.

### 5.2. TR2/TR4 in β-Thalassemia

β-thalassemia arises from insufficient β-globin synthesis, leading to ineffective erythropoiesis and severe anemia. In this context, HbF serves as a compensatory mechanism by partially substituting for deficient HbA, thereby ameliorating clinical manifestations [113,114]. Distinct from SCD, therapeutic management of β-thalassemia necessitates a dual approach: augmenting HbF production while simultaneously addressing impaired erythroid maturation—a critical differentiation in treatment strategy [115].

The TR2/TR4 regulatory axis plays a paradoxical dual role in disease pathogenesis. Primarily, TR2/TR4 heterodimers enforce γ-globin suppression through epigenetic silencing of HBG genes via BCL11A recruitment, thereby constraining HbF’s compensatory potential [71]. However, emerging evidence reveals their secondary involvement in late-stage erythroid differentiation, where they potentially coordinate with transcription factors such as GATA1 to regulate erythroblast enucleation and hemoglobinization processes [10]. This functional dichotomy presents unique therapeutic opportunities: pharmacological inhibition of TR2/TR4 disrupts BCL11A-mediated HBG repression to directly enhance γ-globin synthesis, while targeted modulation of TR4-GATA1 interactions could concurrently optimize erythroid maturation efficiency.

Gene therapy has emerged as a transformative frontier beyond conventional allogeneic transplantation. Advanced modalities employing CRISPR-Cas9-mediated BCL11A knockdown [101,116,117] or engineered disruption of TR2/TR4-DNA binding demonstrate precise HbF reactivation in hematopoietic stem cells [71]. Parallel strategies utilizing lentiviral vectors to deliver modified γ-globin variants [118] or TR2/TR4 decoy elements further exemplify this paradigm. Preclinical evaluations report sustained HbF elevation exceeding 30%, accompanied by normalized erythrocyte indices and reduced hemolysis markers—outcomes that starkly contrast with the transient benefits and substantial morbidity associated with chronic transfusion regimens [119]. By circumventing recurrent treatment costs and transfusion-related complications, these genetic interventions herald a potential curative pathway for β-thalassemia, positioning molecular targeting of the TR2/TR4-BCL11A axis as a cornerstone of next-generation therapeutics.

### 5.3. TR2/TR4 in HPFH

Hereditary persistence of fetal hemoglobin is a clinically significant condition characterized by the continued synthesis of fetal γ-globin in adult erythroid cells, which normally switch to adult β-globin expression [120]. HPFH arises from mutations that impair the developmental silencing of γ-globin, such as the −117 Aγ HPFH variant disrupting a DR1 element in the γ-globin promoter, reducing TR2/TR4 binding affinity by ~3-fold in vitro, mirroring its in vivo effect of γ-globin derepression [11]. Genetic ablation of TR2/TR4 in mice similarly delays fetal-to-adult globin switching, elevating ε/γ-globin transcripts via transcriptional activation. Conversely, forced TR2/TR4 overexpression in erythroid cells suppresses murine embryonic εγ-globin expression, confirming their role as direct repressors [11].

Targeting TR2/TR4-mediated repression offers a strategic avenue for reactivating HbF in β-hemoglobinopathies. As nuclear orphan receptors with ligand-binding domains, TR2/TR4 are druggable candidates for small-molecule inhibitors that disrupt their heterodimerization, DNA binding, or interactions with co-repressors. Alternatively, CRISPR-based epigenetic editing could selectively abrogate DRED complex occupancy at γ-globin promoters, mimicking HPFH-associated derepression. Such interventions would exploit the mechanistic parallels between HPFH mutations and TR2/TR4 inactivation, leveraging endogenous γ-globin activation to ameliorate sickle cell disease or β-thalassemia while avoiding pleiotropic effects. This dual focus on mechanistic elucidation and translational innovation positions TR2/TR4 as pivotal regulators and promising therapeutic targets in hemoglobin switching disorders.

## 6. TR2/TR4-Targeted Coregulators

**Figure 5:**
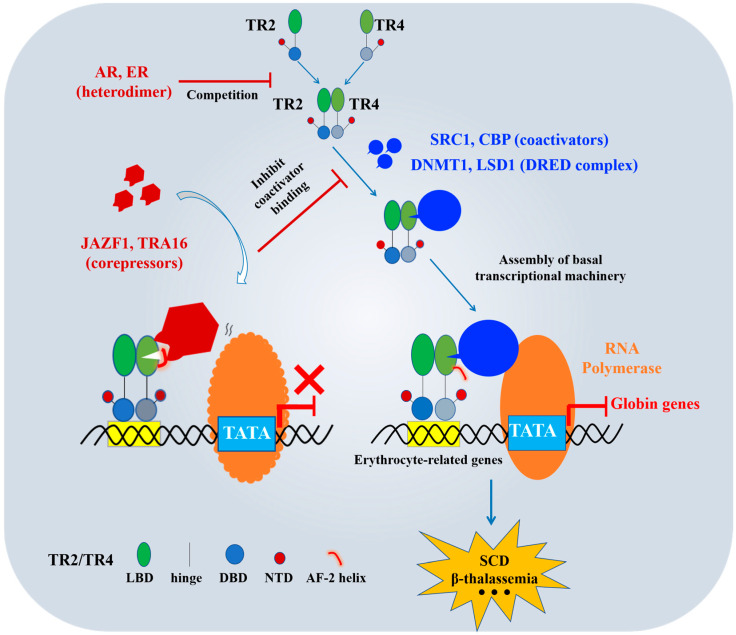
Regulatory diagram of TR2/TR4 coregulators.

TR2/TR4 play pivotal roles in erythroid disorders [42]. These nuclear receptors suppress γ-globin expression by interacting with corepressors within the DRED complex, including DNMT1 and LSD1. Notably, genetic ablation of TR2/TR4 reduces adult β-globin expression while reactivating fetal ε-globin and γ-globin genes, demonstrating their dual regulatory function in orchestrating the developmental globin switch during erythroid maturation [42]. This dual role underscores the therapeutic significance of developing selective TR2/TR4 modulators (inhibitors/activators). Beyond established DRED components (e.g., DNMT1 and LSD1), we have systematically cataloged additional critical TR2/TR4 coregulators (Figure 5).

The transcriptional activity of TR2/TR4 depends critically on coactivator recruitment. Key partners include steroid receptor coactivator 1 (SRC1) [121] and CREB-binding protein (CBP) [122]. SRC1, a member of the p160 coactivator family, binds specifically to LBDs of TR2/TR4 through its conserved nuclear receptor-binding domain (NR box) [123]. This interaction stabilizes receptor-DNA binding and facilitates recruitment of the basal transcriptional machinery, including RNA polymerase II and mediator complexes [124] (Figure 5). Concurrently, CBP exerts its histone acetyltransferase (HAT) activity [125] by acetylating histone H3K27 and H4K16 residues, thereby relaxing chromatin architecture and promoting assembly of the transcription preinitiation complex [126]. For instance, in hepatocytes, TR4 coordinates with SRC1 and CBP to regulate lipid homeostasis through this synergistic mechanism [127]. Such combinatorial regulation not only reveals the context-dependent plasticity of TR2/TR4-mediated transcription but also identifies coactivator complexes as actionable targets for designing therapies against erythroid pathologies [128].

JAZF1, a TR4-specific inhibitor, exhibits potential inhibitory effects on TR2 while demonstrating no activity against other nuclear receptors [129]. Mechanistically, TR4 recruits coactivators such as SRC1 and CBP through its LBD, where these factors bind surfaces typically occupied by its AF-2 helix. JAZF1 disrupts this process by targeting a novel binding surface on TR4, stabilizing a unique α13 helix conformation—a structural feature unreported in other NR family members. This interaction locks the AF-2 helix in an inactive conformation, sterically blocking coactivator recruitment and abolishing TR4’s transcriptional activity [29] (Figure 5). Mutagenesis of key residues in these regions impairs TR4’s interactions and transcriptional activation across experimental models [29,129], providing a structural blueprint for the therapeutic targeting of TR4-associated diseases, including erythroid disorders, diabetes, and malignancies [130].

TRA16, another TR4-specific corepressor, suppresses TR2/TR4 transcriptional activation in a dose-dependent manner [131,132]. Studies by Yang et al. [131] reveal its nuclear localization and tripartite inhibitory mechanism: (1) reducing TR4 binding to TR4 response elements (TR4REs), (2) inhibiting TR4 dimerization, and (3) interfering with interactions between TR4’s DBD and LBD. Given TR4’s established role in repressing γ-globin expression in erythrocytes [10,11], TRA16 overexpression could counteract this suppression, potentially reactivating γ-globin synthesis and fetal HbF production. Such an approach holds promise for restoring normal erythropoiesis and hemoglobin profiles in β-hemoglobinopathies.

The TR2/TR4 regulatory network exhibits complex cross-talk with other NRs (Figure 5). The androgen receptor (AR) forms heterodimers with TR4, mutually repressing each other’s target genes through competitive DNA-binding interference [133]. Similarly, estrogen receptor (ER) engages in reciprocal transcriptional repression via heterodimerization with TR2/TR4 [134]. Strikingly, retinoid X receptor (RXR)—despite being the most ubiquitous NR heterodimer partner—shows no interaction with TR2/TR4, underscoring the specificity of these regulatory circuits [135]. These findings collectively delineate a sophisticated interplay between TR2/TR4 and other NRs, offering multiple entry points for therapeutic intervention while highlighting the need for pathway-selective drug design.

## 7. Limitations

Although TR2 and TR4 have historically been regarded as functionally redundant in erythroid differentiation, emerging evidence reveals their distinct roles during embryonic development and lineage-specific erythroid maturation [10,11,16,91]. However, allogeneic hematopoietic stem cell transplantation [136] remains the only curative intervention, with investigational therapies including erythropoiesis-modulating agents [137] and stem cell-based gene therapies [138] under clinical evaluation. While TR2/TR4 offer novel therapeutic prospects, the genetic or pharmacological manipulation of these receptors necessitates precise control to mitigate off-target effects and ensure lineage-specific outcomes.

As critical orphan nuclear receptors, the identification and characterization of TR2/TR4-specific inhibitors not only advance fundamental mechanistic insights but also unlock clinical opportunities. Nevertheless, translating these discoveries into therapeutic applications faces substantial challenges, including optimizing target specificity, ensuring long-term safety, and achieving durable efficacy in human pathophysiology.

High-resolution structural data are pivotal for elucidating TR2/TR4 functionality, yet current structural models remain fragmentary, with structure–function relationships incompletely resolved. Comprehensive compound screening coupled with mechanistic studies may enable the development of precision-targeted therapeutic strategies against TR2/TR4, potentially revolutionizing treatment paradigms for erythroid disorders. Despite their promise as drug targets, TR2/TR4-directed drug development confronts persistent hurdles, such as achieving subtype selectivity, high binding affinity, and pharmacokinetic stability. Current preclinical research relies heavily on in vitro cell culture systems and transgenic animal models, which may inadequately recapitulate human disease states, thereby limiting the clinical translatability of findings.

## 8. Conclusions

The orphan nuclear receptors TR2/TR4 suppress the developmentally timed expression of embryonic and fetal globin genes through an epigenetic regulatory network. These receptors specifically bind DR elements within the promoter regions of the β-globin gene cluster, recruiting corepressors such as LSD1 and DNMT1 to assemble multiprotein complexes (e.g., the DRED complex), thereby enforcing stage-specific silencing of target genes. Studies demonstrate that functional loss of TR2/TR4 leads to aberrant reactivation of HBE1 and fetal βh1-globin (homologous to HBG1/2), concomitant with impaired terminal erythroid maturation [10,11,16,51,52,53,54,55].

This regulatory mechanism unveils novel therapeutic avenues for hemoglobinopathies: Inhibiting TR2/TR4 activity or targeting their binding sites can reactivate HbF, alleviating erythroid pathological phenotypes in SCD [71,122]. Current advancements using CRISPR-Cas9-mediated editing of HBG1/HBG2 promoter regions have achieved significant HbF elevation in SCD patients. Furthermore, small-molecule modulators targeting TR2/TR4 (e.g., HDAC inhibitors) enhance HbF expression by 2- to 3-fold in preclinical models [17,136]. These breakthroughs underscore the therapeutic potential of precision modulation of the TR2/TR4 complex’s epigenetic activity, offering a causal intervention to ameliorate the pathological progression of hemoglobinopathies.

## Data Availability

Not applicable.
